# A novel microbe-drug association prediction model based on graph attention networks and bilayer random forest

**DOI:** 10.1186/s12859-024-05687-9

**Published:** 2024-02-20

**Authors:** Haiyue Kuang, Zhen Zhang, Bin Zeng, Xin Liu, Hao Zuo, Xingye Xu, Lei Wang

**Affiliations:** Big Data Innovation and Entrepreneurship Education Center of Hunan Province, Changsha University, Changsha, 410022 China

**Keywords:** Graph attention networks, Bilayer random forest, Microbial-drug networks, Contribution value

## Abstract

**Background:**

In recent years, the extensive use of drugs and antibiotics has led to increasing microbial resistance. Therefore, it becomes crucial to explore deep connections between drugs and microbes. However, traditional biological experiments are very expensive and time-consuming. Therefore, it is meaningful to develop efficient computational models to forecast potential microbe-drug associations.

**Results:**

In this manuscript, we proposed a novel prediction model called GARFMDA by combining graph attention networks and bilayer random forest to infer probable microbe-drug correlations. In GARFMDA, through integrating different microbe-drug-disease correlation indices, we constructed two different microbe-drug networks first. And then, based on multiple measures of similarity, we constructed a unique feature matrix for drugs and microbes respectively. Next, we fed these newly-obtained microbe-drug networks together with feature matrices into the graph attention network to extract the low-dimensional feature representations for drugs and microbes separately. Thereafter, these low-dimensional feature representations, along with the feature matrices, would be further inputted into the first layer of the Bilayer random forest model to obtain the contribution values of all features. And then, after removing features with low contribution values, these contribution values would be fed into the second layer of the Bilayer random forest to detect potential links between microbes and drugs.

**Conclusions:**

Experimental results and case studies show that GARFMDA can achieve better prediction performance than state-of-the-art approaches, which means that GARFMDA may be a useful tool in the field of microbe-drug association prediction in the future. Besides, the source code of GARFMDA is available at https://github.com/KuangHaiYue/GARFMDA.git

**Supplementary Information:**

The online version contains supplementary material available at 10.1186/s12859-024-05687-9.

## Background

A multitude of microbial communities, including bacteria, fungi, viruses, and other microbes, have been found in the human body, which are intimately linked to human health and are crucial to numerous physiological processes, including immune regulation, vitamin production, and the maintenance of digestive function [1, 2]. However, some microorganisms may be associated with the development of disease under specific circumstances. For instance, an imbalance of human gut bacteria can lead to the risk of high blood pressure [3].

In recent years, the misuse and irrational use of antibiotics, mutation and horizontal gene transfer of microbial genes, and the spread of microorganisms in the medical and social environments have led to microbial resistance to antibiotics, which makes effective antibiotic treatment ineffective and poses a serious challenge to clinical treatment [4]. Therefore, in order to address the problem of microbial resistance, it is meaningful to develop efficient computational models to detect microbial resistance and find new antibiotics, because these computational models can infer latent microbe-drug associations and thus provide a simple and efficient way to address microbial resistance.

For the last few years, a number of databases of microbial-drug associations, including MDAD [5], aBiofilm [6], and Drugvirus [7], have been adopted by researchers to construct an abundance of calculation models to identify possible microbe-drug associations. For example, in 2019, Zhu et al. [8] created a prediction model named HMDAKATZ based on the KATZ measure. In 2021, Deng et al. [9] devised a method called Graph2MDA by constructing multimodal attribute graphs as inputs of variogram autoencoders to discover details about every node and the complete graph. Long et al. [10] introduced the metapath2vec scheme for learning low-dimensional embedded representations of microorganisms and drugs and designed a partial dichotomous network projection recommendation algorithm and proposed a novel calculation method named HNERMDA. In 2023, Ma et al. [11] combined graph attention networks and CNN-based classifiers to construct a model called GACNNMDA. Huang et al. [12] designed a model named GNAEMDA based on graph normalized convolutional networks. Cheng et al. [13] designed a model called NIRBMMDA based on the neighbourhood-based inference and the restricted Boltzmann machine. Li et al. [14] combined matrix decomposition and a three-layer heterogeneous network to create a model called MFTLHNMDA to infer microbe-drug associations.

In this article, in order to improve the performance of prediction models, we designed a new prediction model named GARFMDA by combining graph attention network (GAT) and two-layer random forest (RF). In GARFMDA, a two-layer GAT was adopted first to learn the low-dimensional feature representations of microbes and drugs. And then, a two-layer random forest model was introduced to obtain the contribution values of all features as well as predict possible associations between microorganisms and drugs after eliminating those low-contribution features. Additionally, we conducted extensive case studies and comparison experiments to assess the prediction performance of GARFMDA. And as a result, GARFMDA achieved satisfactory results in the field of possible microbe-drug relationship prediction and outperformed existing representative competing methods.

### Data sources

In this section, we will first download known microbe-drug associations from the MDAD database (https://figshare.com/search?q=10.6084%2Fm9.figshare.24798456), which consists of 2470 validated microbe-drug associations, including 1373 drugs and 173 microbes. Subsequently, we will download additional data on microbe, drug and disease associations from the database proposed by Wang et al. [14], which contains 70,315 reported drug-disease connections and 15,633 reported microbe-disease connections. Following a rigorous screening procedure to eliminate disease-related correlations for which there is no known association between medications or microorganisms in the MDAD database, we finally obtain 109 unique drug-disease connections covering 1,121 drugs and 233 diseases, and 109 unique microbe-disease connections covering 402 microbes and 73 diseases from the database proposed by Wang et al. Furthermore, we have also gathered 138 known microbe-microbe interactions, encompassing 123 microbe in MDAD, and 5586 known drug-drug relationships, from the data collection created by Deng et al. [9], which covers 1228 drugs in MDAD. Additional files 1, 2, 3, 4, 5, 6, 7, 8 and Table 1 below provides information on the aforementioned facts.

**Table 1:** Specifics of the newly-downloaded dataset

Type	Associations	Microbes	Drugs	Disease
Microbe-disease associations	402	73	–	109
Microbe-drug associations	2470	173	1373	–
Drug-disease associations	1121	–	233	109
Drug-drug interactions	5586	–	1228	–
Microbe-microbe interactions	138	123	–	–

## Methods

**Fig.1:**
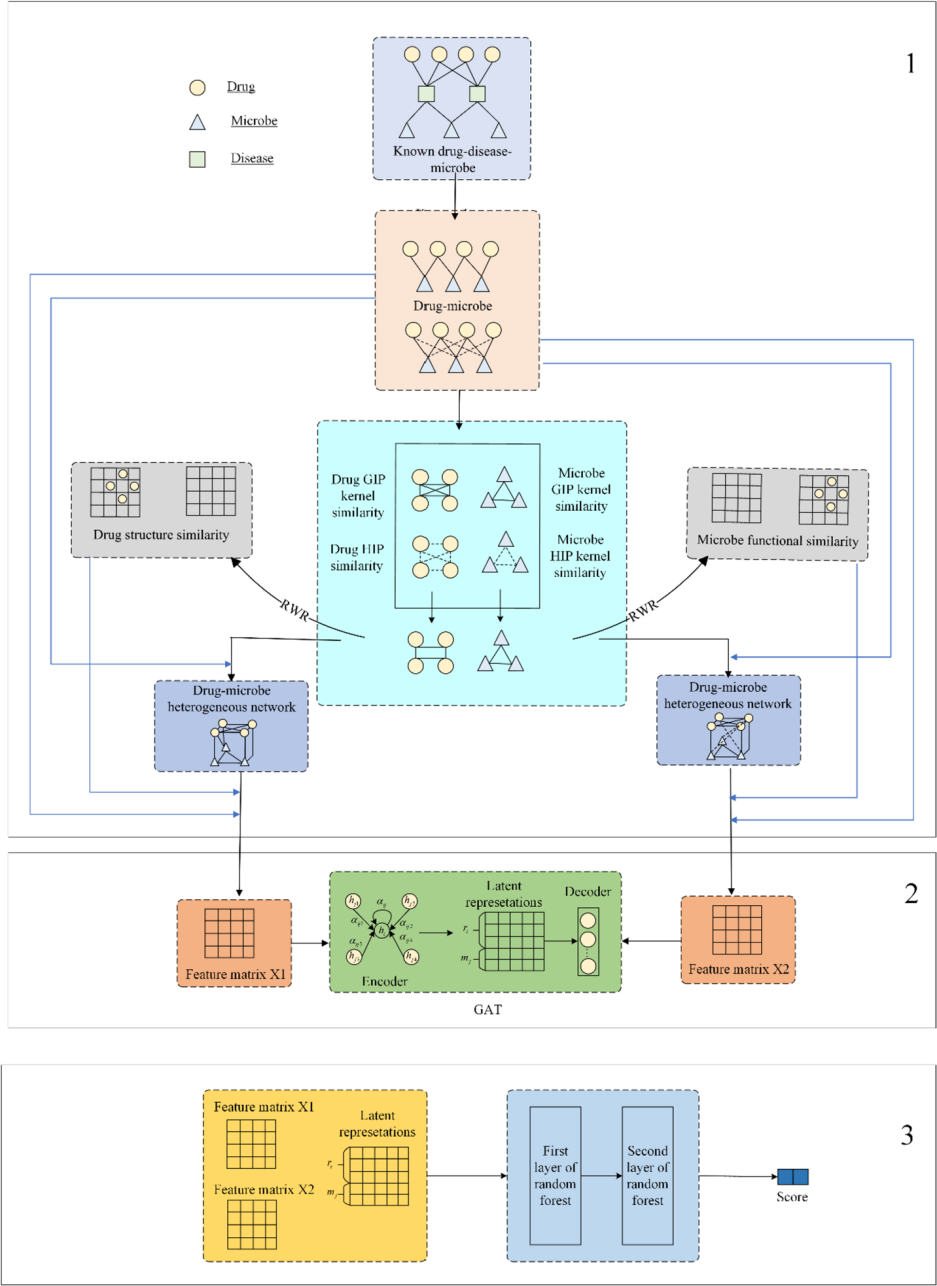
Flowchart of GARFMDA

As shown in Fig. 1, GARFMDA is composed of the following three main parts:

*Part 1*: Firstly, based on the newly-downloaded datasets on microbes, drugs and diseases, two different heterogeneous microbe-drug networks $H{N}_{1}$ and $H{N}_{2}$ will be constructed.

*Part 2*: And then, based on multiple similarity metrics of microbe and drug, a feature matrix will be created for microbes and drugs separately, which will be then fed into the GAT along with $H{N}_{1}$ and $H{N}_{2}$ to learn the low-dimensional feature representations for microbes and drugs respectively.

*Part 3*: Finally, these two newly-obtained low-dimensional feature representations, along with two feature matrices, will be inputted into a two-layer random forest model to compute the probability scores of drug-microbe relationships.

### Construction of two heterogeneous microbe-drug networks

For any given database *D*, let ${n}_{r}$ and ${n}_{m}$ stand for the numbers of drugs and microorganisms newly downloaded from *D* respectively, then we can construct a adjacency matrix ${D}^{1}\in {R}^{{n}_{r}*{n}_{m}}$ between microbes and drugs as follows: for any given microbe ${m}_{j}$ and drug ${r}_{i}$, if there is a known relationship between them in *D*, there is ${D}^{1}\left(i,j\right)=1$, otherwise there is ${D}^{1}\left(i,j\right)=0$.

Similarly, based on the newly-downloaded datasets of known connections between microbes and drugs, microbes and diseases, and drugs and diseases, we may create another microbe-drug adjacency matrix ${D}^{2}\in {R}^{{n}_{r}*{n}_{m}}$ as follows: for a given microbe ${m}_{j}$, drug ${r}_{i}$ and disease ${d}_{k}$, if there exist a known relationship between ${m}_{j}$ and ${d}_{k}$, as well as a known association between ${r}_{i}$ and ${d}_{k}$, then there is ${D}^{2}\left(i,j\right)=1$, otherwise there is ${D}^{2}\left(i,j\right)=0$.

Hence, based on above two adjacency matrices ${D}^{1}$ and ${D}^{2}$, it is simple to build two heterogeneous microbe-drug networks $H{N}_{1}$ and $H{N}_{2}$ according to the following way:

Firstly, in ${D}^{v}\left(v=\mathrm{1,2}\right)$, let ${D}^{v}\left({r}_{i}\right)$ and ${D}^{v}\left({m}_{j}\right)$ denote the *i*-th row and* j*-th column of ${D}^{v}$ separately, then for any two given drugs ${r}_{i}$ and ${r}_{j}$, we will calculate the Gaussian Interaction Profile (GIP) kernel similarity between them as follows:

1 $${A}_{rg}^{v}\left({r}_{i},{r}_{j}\right)= exp\left(-{\gamma }^{1}\| {D}^{v}\left({r}_{i}\right)- {D}^{v}\left({r}_{j}\right){\| }^{2}\right)$$

2 $${\gamma }^{1}=1/\left(\frac{1}{{n}_{r}}\sum_{i=1}^{{n}_{r}}\| {D}^{v}\left({r}_{i}\right){\| }^{2}\right)$$

where ‖·‖ denotes the Frobenius norm.

Obviously, based on above Eq. (1), we can obtain a GIP kernel similarity matrix ${A}_{rg}^{v}\in {R}^{{n}_{r}*{n}_{r}}$ for drugs.

In a similar way, for any two given microbes ${m}_{i}$ and ${m}_{j}$, we can also calculate the GIP kernel similarity between them as follows:

3 $${A}_{mg}^{v}\left({m}_{i},{m}_{j}\right)= exp\left(-{\gamma }^{2}\| {D}^{v}\left({m}_{i}\right)- {D}^{v}\left({m}_{j}\right){\| }^{2}\right)$$

4 $${\gamma }^{2}=1/\left(\frac{1}{{n}_{m}}\sum_{i=1}^{{n}_{m}}\| {D}^{v}\left({m}_{i}\right){\| }^{2}\right)$$

Obviously, based on above Eq. (3), we can obtain a GIP kernel similarity matrix ${A}_{mg}^{v}\in {R}^{{n}_{m}*{n}_{m}}$ for microbes as well.

Next, based on the assumption that when two nodes have highly dissimilar interaction characteristics, they are less comparable to each other [15], for any two given drugs ${r}_{i}$ and ${r}_{j}$, we will calculate the Hamming Interaction Profile (HIP) similarity between them as follows:

5 $${A}_{rh}^{v}\left({r}_{i},{r}_{j}\right)=1-\frac{|{D}^{v}\left({r}_{i}\right)!={D}^{v}\left({r}_{j}\right) |}{|{D}^{v}\left({r}_{i}\right)|}$$

Here, |${D}^{v}\left({r}_{i}\right)$| represents the number of elements in ${D}^{v}\left({r}_{i}\right)$, and $|{D}^{v}\left({r}_{i}\right)!={D}^{v}\left({r}_{j}\right) |$ indicates the number of distinct elements between ${D}^{v}\left({r}_{i}\right)$ and ${D}^{v}\left({r}_{j}\right)$.

Similarly, for any two given microbe ${m}_{i}$ and ${m}_{j}$, the HIP similarity between them can be determined as follows:

6 $${A}_{mh}^{v}\left({m}_{i},{m}_{j}\right)=1-\frac{|{D}^{v}\left({m}_{i}\right)!={D}^{v}\left({m}_{j}\right) |}{|{D}^{v}\left(m\right)|}$$

Here, $|{D}^{v}\left({m}_{i}\right)!={D}^{v}\left({m}_{j}\right) |$ indicates the number of distinct elements between ${D}^{v}\left({m}_{i}\right)$ and ${D}^{v}\left({m}_{j}\right)$, and $|{D}^{v}\left(m\right)|$ denotes the number of elements in ${D}^{v}\left(m\right)$.

Hence, based on above Eqs. (5) and (6), we can obtain two HIP similarity matrices ${A}_{rh}^{v}\in {R}^{{n}_{r}*{n}_{r}}$ and ${A}_{mh}^{v}\in {R}^{{n}_{m}*{n}_{m}}$ for drugs and microbes separately.

Finally, for any two given drugs ${r}_{i}$ and ${r}_{j}$, it is evident that we can construct an integrated similarity between them by integrating ${A}_{rg}^{v}$ and ${A}_{rh}^{v}$ as follows:

7 $$A_{r}^{v} \left( {r_{i} ,r_{j} } \right) = \left\{ {\begin{array}{*{20}l} {1: } \hfill & {{\text{if there is a known association between}}\; r_{i} \;{\text{and }}\;r_{j} } \hfill \\ {\frac{{A_{rg}^{v} \left( {r_{i} ,r_{j} } \right) + A_{rh}^{v} \left( {r_{i} ,r_{j} } \right)}}{2}: } \hfill & {\text{ therwise}} \hfill \\ \end{array} } \right.$$

Similarly, for any two given microbes ${m}_{i}$ and ${m}_{j}$, we can construct an integrated similarity between them by integrating ${A}_{mg}^{v}$ and ${A}_{mh}^{v}$ as follows:

8 $$A_{m}^{v} \left( {m_{i} ,m_{j} } \right) = \left\{ {\begin{array}{*{20}l} {1 :} \hfill & { {\text{if there is a known association between}}\; m_{i} \; {\text{and}}\; m_{j} } \hfill \\ {\frac{{A_{mg}^{v} \left( {m_{i} ,m_{j} } \right) + A_{mh}^{v} \left( {m_{i} ,m_{j} } \right)}}{2}:} \hfill & {{\text{otherwise}}} \hfill \\ \end{array} } \right.$$

Hence, based on above Eqs. (7) and (8), we can finally obtain two new matrices ${H}^{1}\in {R}^{\left({n}_{r}+{n}_{m}\right)*\left({n}_{r}+{n}_{m}\right)}$ and ${H}^{2}\in {R}^{\left({n}_{r}+{n}_{m}\right)*\left({n}_{r}{+n}_{m}\right)}$ as follows:

9 $${H}^{1}=\left[\begin{array}{cc}{A}_{r}^{1}& {D}^{1}\\ {\left({D}^{1}\right)}^{T}& {A}_{m}^{1}\end{array}\right]$$

10 $${H}^{2}=\left[\begin{array}{cc}{A}_{r}^{2}& {D}^{2}\\ {\left({D}^{2}\right)}^{T}& {A}_{m}^{2}\end{array}\right]$$

Obviously, based on the above two matrices *H*^*1*^ and *H*^*2*^, two heterogeneous microbe-drug networks ${{\text{HN}}}_{1}$ and ${{\text{HN}}}_{2}$ can be constructed respectively.

### Extracting low-dimensional feature representations for microbes and drugs by GAT

#### Constructing unique feature matrix for microbes and drugs

In this section, we will first adopt the SIMCOMP2 [16] to determine the structural similarity between any two given drugs ${r}_{i}$ and ${r}_{j}$, and obtain a new drug structural similarity matrix ${A}_{rc}$. Next, we will utilize the method presented by Kamneva [17] to determine the functional similarity between any two given microorganisms ${m}_{i}$ and ${m}_{j}$, and create a new microbe functional similarity matrix ${A}_{mf}$. And then, we will further perform RWR [39] on ${A}_{r}^{v}$ and ${A}_{m}^{v}$ separately in the following way:

11 $${q}_{i}^{l+1}=\lambda Q{q}_{i}^{l}+(1-\lambda ){\beta }_{i}$$

In above equations, *Q* is the matrix of transition probabilities, ${q}_{i}^{l}$ is the likelihood of node *i* transferring to the node* l*, and ${\beta }_{i}\in {R}^{1*n}$ is the starting odds vector for the node *i*, and the *j*-th element in ${\beta }_{i}$ is defined as follows:

12 $$\beta_{i,j} = \left\{ {\begin{array}{*{20}l} {1:} \hfill & {{\text{if}}\; i = j} \hfill \\ {0:} \hfill & {otherwise} \hfill \\ \end{array} } \right.$$

Obviously, based on above Eqs. (11) and (12), we can obtain two different matrices ${A}_{rr}^{v}$ and ${A}_{mm}^{v}$ based on ${A}_{r}^{v}$ and ${A}_{m}^{v}$ respectively.

Thereafter, based on above newly obtained matrices, we can construct a unique feature matrix to preserve more original features of microbes and drugs as follows:

13 $${S}^{v}=\left[\begin{array}{c}{F}_{r}^{v}\\ {F}_{m}^{v}\end{array}\right]$$

where,

14 $${F}_{r}^{v}= \left[{A}_{rc};{D}^{v};{A}_{rr}^{v};{D}^{v}\right]$$

15 $${F}_{m}^{v}=\left[{\left({A}^{v}\right)}^{T};{S}_{mf};{\left({A}^{v}\right)}^{T};{S}_{mm}^{v}\right]$$

From above Eqs. (13), (14) and (15), it is clear that there is ${S}^{v}\in {R}^{\left({n}_{r}+{n}_{m}\right)*{k}_{1}}$ $\left(v=\mathrm{1,2}\right)$, where, *k*_1_ represents the number of columns in ${S}^{v}$.

#### The structure of the two-layer GAT

*Encoder*: To determine the degree of similarity between any given node *i* and one of its neighboring node *j* in ${H}^{v}\left(v=\mathrm{1,2}\right)$, we will compute the similarity coefficient ${e}_{ij}$ between them as follows:

16 $${e}_{ij}= LeakyRelu\left(\alpha \left[{W}^{v}{S}^{v}\left(i\right);{W}^{v}{S}^{v}\left(j\right)\right]\right),j\in {\varphi }_{i}^{v}$$

17 $$LeakyRelu\left( x \right) = \left\{ {\begin{array}{*{20}l} x \hfill & {x > 0} \hfill \\ {\mu x} \hfill & {otherwise} \hfill \\ \end{array} } \right.$$

where ${S}^{v}\left(i\right)$ denotes the *i*-th row of ${S}^{v}$, $\alpha$ is an operation for feature mapping, ${W}^{v}$ is a trainable weight matrix, ${\varphi }_{i}^{v}$ is the collection of nodes that are adjacent to *i* in ${H}^{v}$, and $\mu$ is a hyper-parameter varying between 0 and 1.

Based on above Eq. (16), for any two given nodes *i* and *j*, then the attention score ${\rho }_{ij}$ between them can be calculated as follows:

18 $${\rho }_{ij}= \frac{exp\left({e}_{ij}\right)}{\sum_{k\in {\varphi }_{i}^{v}}exp\left({e}_{ik}\right)}$$

Obviously, based on above attention score ${\rho }_{ij}$, a new feature of node *i*, representing the weighted sum of the features of its neighboring nodes, can be obtained as follows:

19 $${M}^{v}\left(i\right)=Relu\left(\sum_{j\in {\varphi }_{i}^{v}}{\rho }_{ij}{W}^{v}{S}^{v}\left(j\right)\right)$$

20 $$Relu\left( x \right) = \left\{ {\begin{array}{*{20}l} x \hfill & {x > 0} \hfill \\ 0 \hfill & {otherwise} \hfill \\ \end{array} } \right.$$

Hence, we can construct a new feature representation matrix ${M}^{v}$ as follows:

21 $${M}^{v}=\left[\begin{array}{c}{R}_{r}^{v}\\ {R}_{m}^{v}\end{array}\right]\in {R}^{\left({n}_{r}+{n}_{m}\right)*{k}_{2}}$$

Here, ${k}_{2}$ represents the nunber of columns in ${M}^{v}$.

*Decoder*: Te decoder adopts the same structure as the encoder, and is defined as follows::

22 $$M^{\prime v} = sigmoid\left( {M^{v} \cdot \left( {M^{v} } \right)^{T} } \right)$$

23 $$sigmoid\left(x\right)= \frac{1}{1+{e}^{-x}}$$

*Optimization*: Taking into account the fact that the reconstructed matrix differs from the raw matrix, we adopt the MSE loss factor to determine the average of the sum of differences squared between $M^{\prime v}$ and $H^{v}$. The MSE loss function is defined as follows:

24 $$Loss = \frac{1}{{n_{r} + n_{m} }}\mathop \sum \limits_{i = 1}^{{n_{r} + n_{m} }} M^{\prime v} \left( i \right) - H^{v} \left( i \right)^{2}$$

where $M^{\prime v} \left( i \right)$ and $H^{v} \left( i \right)$ denote the *i*-th row of $M^{\prime v}$ and ${H}^{v}$ respectively.

Finally, Finally, the Adam optimizer [40] will be further used to optimize the loss function in the model training process.

**Fig. 2:**
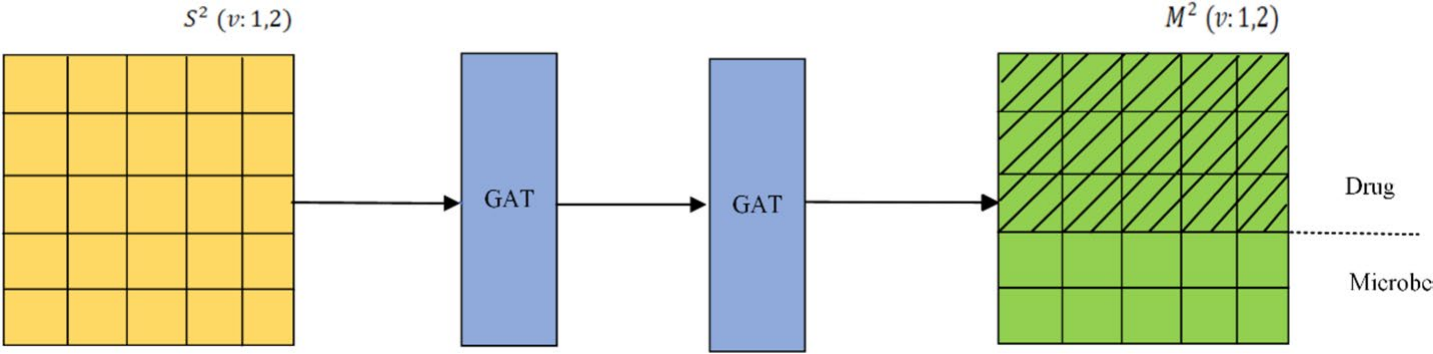
workflow of the two-layer GAT in GARFMDA

Furthermore, we present the workflow of the two-layer GAT in the following Fig. 2 for better understanding the implementation of the above two-layer GAT.

### The structure of the two-layer random forest

Traditional machine learning, when faced with complex nonlinear patterns, may suffer from drawbacks such as overfitting problems and the inability to provide uncertainty estimates of the predicted outcomes [18]. In order to calculate the potential scores of unknown drug-microbe relationships, we will create a two-layer random forest model in this section and treat the drug-microbe problem as a binary classification problem, which can improve the model effect and reduces the risk of overfitting through the selection of features in the first layer of the random forest. For the input of the first layer of the two-layer random forest, we will respectively construct two feature matrices ${B}_{r}^{v}$ and ${B}_{m}^{v}$ according to the following equations:

25 $${B}_{r}^{v}= \left[{R}_{r}^{v}; {F}_{r}^{v}\right]$$

26 $${B}_{m}^{v}= \left[{R}_{m,}^{v};{F}_{m}^{v}\right]$$

And then, for any given drug ${r}_{i}$ and microbe ${m}_{j}$, let ${B}_{r}^{v}\left(i\right)$ and ${B}_{m}^{v}\left(j\right)$ represent the *i-*th row of ${B}_{r}^{v}$ and the *j*-th column of ${B}_{m}^{v}$ respectively, and $${F}^{v}\left(i,j\right)= \left[\begin{array}{c}{B}_{r}^{v}\left(i\right)\\ {B}_{m}^{v}\left(j\right)\end{array}\right]\in {R}^{\left({n}_{r}\times {n}_{m}\right)*2*{k}_{3}}$$, where *k*_3_ represents the number of columns in ${F}^{v}$, then we will feed ${F}^{v}$ into the first layer of the bilayer random forest.

Moreover, in the first layer of the bilayer random forest, we will assume that the number of decision trees is *p* and the maximum depth is *s*. And after training, we will compare the magnitude of the contribution made by each feature during the growth of each decision tree in the bilayer random forest by calculating the sum of the Gini index [19] changes of each feature over all the decision trees in the forest $G\left(tr\right)$ to represent the contribution made by the feature $C\left(tr\right)$, which is defined as follows:

27 $$G\left(tr\right)=\sum Gini\left({F}^{v}\left(tr\right)\right)-Gini\left({F}_{h}^{v}\left(tr\right)\right)$$

28 $$C\left( {tr} \right) = \left( {G\left( {tr} \right)/\sum G\left( k \right)} \right)*100\% , \;\;where \;\;k \in \left( {1,m} \right)$$

where *tr* denotes the feature index, *h* represents the decision tree index, and *m* is the total number of features. $Gini\left({F}_{h}^{v}\left(tr\right)\right)$ denotes the Gini index on the decision tree* h* conditional on the feature *tr*.

After that, we will eliminate the features with contribution value less than *L*, and obtain a new feature matrix ${F}^{{\prime}v}$, which will be fed into the second layer of the bilayer random forest for training and prediction. Hence, we can obtain a score matrix finally.

Obviously, based on the matrices ${H}^{1}$ and ${H}^{2}$, we can obtain two different score matrices ${Score}^{1}$ and ${Score}^{2}$ respectively. Therefore, we can construct an integrated score matrix $S\in {R}^{{n}_{r}*{n}_{m}}$ as follows:

29 $$S\left(i,j\right)= \frac{{Score}^{1}\left(i,j\right)+{Score}^{2}\left(i,j\right)}{2}$$

## Results

In this section, we will first examine the impact of parameters on the prediction performance of GARFMDA. And then, we will compare GARFMDA with five cutting-edge competitive prediction techniques. Finally, in order to illustrate the efficiency of GARFMDA, we will introduce some well-known drugs and microbes for case studies.

### Sensitivity analysis of hyperparameters

From above descriptions, it is clear that there are some important parameters in GARFMDA, including the GAT learning rate, the GAT dropout rate, the maximum depth of the decision tree in the bilayer random forest, and the contribution value of these chosen features. In this section, we will execute 10 times of fivefold Cross Validation (CV) on MDAD to assess impact of these parameters on the effectiveness of GARFMDA for determining the best values of these parameters.

**Fig. 3:**
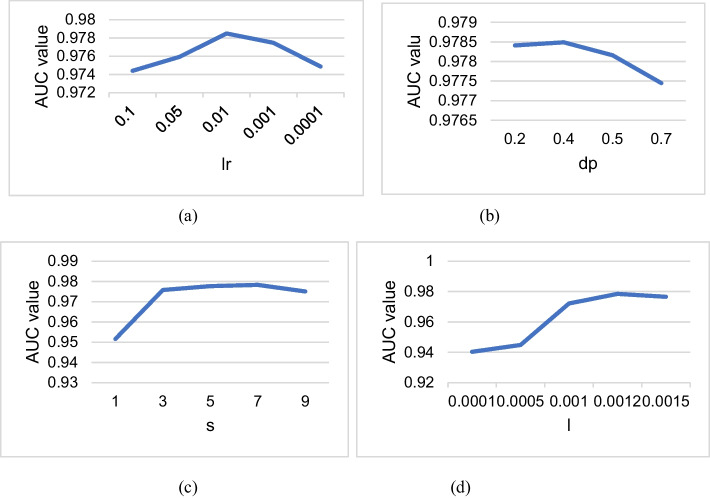
Effects of parameters on performance of GARFMDA. **a** and **b** show the AUC values achieved by GARFMDA with different learning and abandonment rates of GAT, respectively. **c** and **d** illustrate the AUC values achieved by GARFMDA under different maximum depths of decision trees and contribution values of selected features in the bilayer random forest, separately

For simplicity, in experiments, we will use the abbreviations *lr*, *dp*,* s* and *l* to stand for the learning rate and the dropout rate of GAT, the maximum depth of the first and second layers of the decision tree in the bilayer random forest, and the contribution value of these chosen features, respectively. Firstly, we will evaluate the impact of *lr* on the prediction performance of GARFMDA while it varies in the range of {0.0001, 0.001, 0.01, 0.05, 0.1}. From observing the following Fig. 3a, it is clear that when *lr* is set to 0,01, GARFMDA can achieve the highest value of AUC. Next, we will limit the value of *dp* to a range of {0.2, 0.4, 0.5, 0.7}, and as shown in Fig. 3b, it is obvious that when *dp* is set to 0.4, GARFMDA can achieve the highest value of AUC. Additionally, we will restrict the value of *s* to the range of {1, 3, 5, 7, 9} and as illustrated in Fig. 3c, it is evident that when *s* is set to 7, GARFMDA can achieve the highest value of AUC. Finally, we will limit the value of *l* to a range of {0.0001, 0.0005, 0.001, 0.0012, 0.0015}, and as shown in Fig. 3d, the performance of GARFMDA will reach to the best when *l* is set to 0.0012.

As for the parameter *pf* of the number of random forest trees in the bilayer random forest, we found through comparative experiments that the effect of the value of *pf* on the prediction performance of GARFMDA is not significant, but the computational efficiency of GARFMDA will be reduced when *pf* is set to a large number, therefore, we will set the size of decision trees in both layers of the bilayer random forest to 250 during experiments. Similarly, for the parameter of the number of training rounds of GAT, we found through experiments that its numerical size has little effect on the prediction performance of GARFMDA, so we will set it to 10. Furthermore, to make our model better, we will use these parameters that work best to evaluate GARFMDA, i.e., we will set *lr* to 0.01, *dp* to 0.4, *s* to 7 and* l* to 0.0012 in subsequent comparison experiments.

### Comparison with state-of-the-art methods

To validate the predictive performance of GARFMDA, we will compare it with the following five representative approaches separately:

1. LAGCN [20]: which is a computational model for inferring unknown drug-disease associations based on graph convolutional networks and attention mechanisms
2. GSAMDA [21]: which is a microbe-drug association prediction model based on graph attention networks and sparse autoencoders
3. SCSMDA [22]: which aims to predict microbe-drug associations based on the structure-enhanced contrast learning and self-paced negative sampling strategies.
4. MDASAE [23]: which is a calculation method based on fusing multi-attention mechanisms with stacked autoencoders to detect possible microbial drug associations.
5. LRLSHMDA [24]: which is a computational scheme by exploiting Laplace Regularised Least Squares to predict microbe-disease associations.

**Fig. 4:**
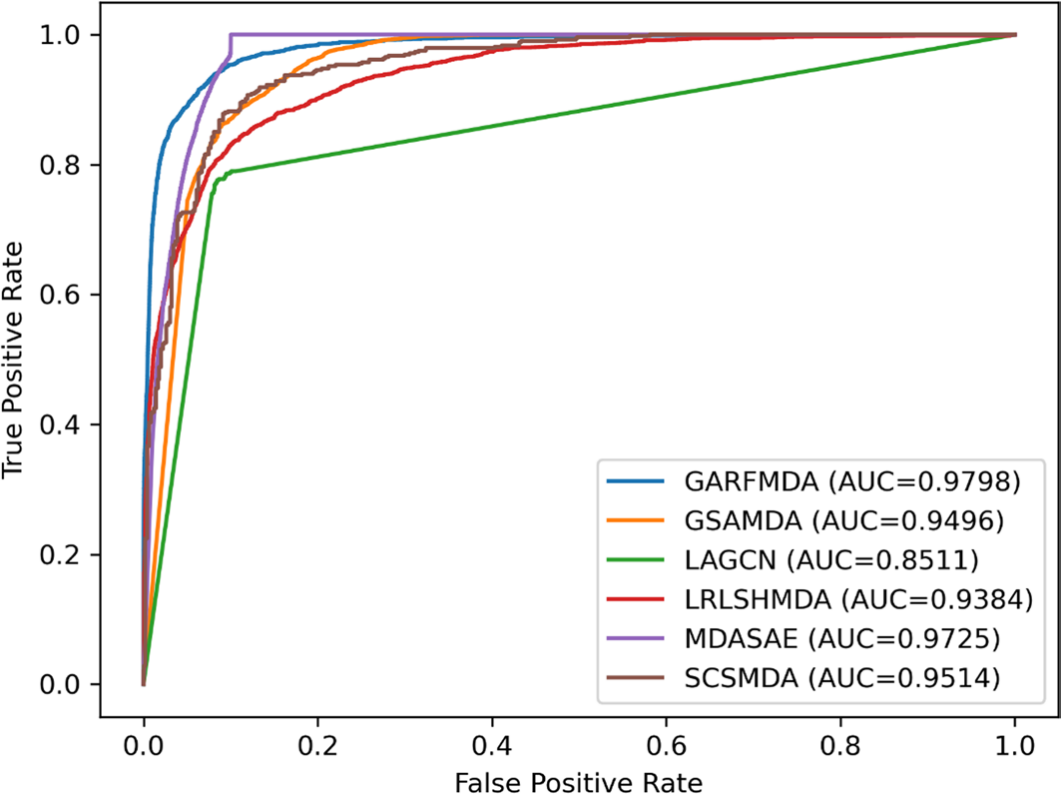
ROC curves achieved by competing techniques on MDAD

During experiments, we will adopt the AUC values, the Accuracy values and the F1-score values as performance indicators and compare all of these rival approaches under the framework of tenfold cross validation. Experimental results are shown in the following Table 2 and Fig. 4 respectively. From observing the Table 2, it is easy to see that GARFMDA can reach to the highest AUC value of 0.9794 ± 0.0012, while MDASAE comes in second with an AUC value of 0.9701 ± 0.0023, and LAGCN has the lowest AUC value of 0.8544 ± 0.0042. As For the Accuracy values and F1-score values, GARFMDA can as well obtain the highest values of 0.9955 and 0.7106 respectively. Therefore, It is obvious that GARFMDA can achieve the best prediction performance among all these five competing models.

**Table 2:** AUC values, Accuracy values and F1-score values obtained by GARFMDA and five competing methods under the framework of tenfold CV on MDAD

Methods	AUC(tenfold)	Accuracy	F1-score
LAGCN	0.8544 ± 0.0042	0.9413	0.1838
GSAMDA	0.9493 ± 0.0003	0.9896	0.6433
MDASAE	0.9701 ± 0.0023	0.9876	0.6959
SCSMDA	0.9546 ± 0.0037	0.9884	0.7016
LRLSHMDA	0.9259 ± 0.0031	0.9365	0.2594
GARFMDA(our model)	0.9794 ± 0.0012	0.9955	0.7106

### Case study

In this section, we will undertake case studies of two well-known medications and one well-known microbe to better illustrate the efficacy of GARFMDA. In experiments, we will choose the top 20 candidate microbes or drugs predicted by GARFMDA and search in PubMed (https://pubmed.ncbi.nlm.nih.gov) for these candidate microbes or drugs to see if any publications had reported about them. Among them, the first drug we have chosen is ciprofloxacin, which is a synthetic second-generation quinolone antimicrobial drug with broad-spectrum antimicrobial activity and bactericidal efficacy, and can be used to treat illnesses caused by mycobacterium influenzae, escherichia coli, and pneumococcus specific polysaccharide [25]. In both vitro and vivo studies of ciprofloxacin, a very low incidence of resistant microorganisms has been reported [26].

In addition, Alhajj et al. [27] developed a dry powder of ciprofloxacin for inhalation for treating cystic fibrosis lung infections. Golapudi et al. demonstrated that ciprofloxacin inhibits TNF-(α)-induced HIV secretion in U1 cells [28]. Table 3 illustrates that there are 19 out of those top 20 predicted potential bacteria having been confirmed by published journals to be related to ciprofloxacin.

**Table 3:** The top 20 predicted candidate ciprofloxacin-associated bacteria. In this table, the first column lists the top 10 predicted microbes, while the third column lists the top 11 to 20 predicted microbes

Microbe	Evidence	Microbe	Evidence
*Streptococcus sanguis*	PMID:8192181	*Fusarium solani*	PMID:19751392
*Stenotrophomonas maltophilia*	PMID:30448331	*Bacteroides fragilis*	PMID:2939556
*Enterococcus faecalis*	PMID:23789048	*Proteus mirabilis*	PMID:27303616
*Firmicutes*	PMID:37047789	*Burkholderia multivorans*	PMID:34524889
*Salmonella Typhi*	PMID:31877141	*Cryptococcus neoformans*	PMID:29858266
*Streptococcus parasanguinis*	PMID:21193474	*Pseudoalteromonas *sp.	PMID:31137680
*Streptococcus mitis*	PMID:10348783	*Halomonas pacifica*	Unconfirmed
*Enterobacter aerogenes*	PMID:22106222	*Pseudomonas japonica*	PMID:30550842
Baker's yeast	PMID:29346617	Hepatitis B virus F	PMID:15365265
*Candida parapsilosis*	PMID:32576753	*Staphylococcus chromogenes*	PMID:17475456

The second drug we have selected is moxifloxacin, a quinolone broad-spectrum antimicrobial that treats adults (≥ 18 years of age) suffering from respiratory tract infections, both upper and lower [29], as well as acute sinusitis [30], acute exacerbations of chronic bronchitis [31], community-acquired pneumonia [32], and skin and soft tissue infections [33]. Januel et al. [34] studied the use of moxifloxacin to treat the genetic disorder spinal muscular atrophy (SMA). However, Inada et al. [35] found that moxifloxacin can induce aortic aneurysms and clips by increasing bone bridging proteins in mice.

Table 4 shows that there are 15 out of the top 20 predicted candidate microorganisms have been confirmed by published journals to be associated with moxifloxacin, demonstrating the value of GARFMDA for clinical drug application and the identification of possible drug-related bacteria.

**Table 4:** The top 20 predicted candidate moxifloxacin-associated bacteria. In this table, the first column lists the top10 predicted microbes, while the third column lists the top 11 to 20 predicted microbes

Microbe	Evidence	Microbe	Evidence
Human respiratory syncytial virus B	PMID:30723301	*Arthrobacter *sp.	PMID:33675087
*Aeromonas hydrophila*	PMID:26588876	*Kocuria rhizophila*	Unconfirmed
*Clostridium leptum*	Unconfirmed	*Porphyromonas gingivalis*	PMID:30048853
*Staphylococcus saprophyticus*	PMID:24982521	Hepatitis C virus	PMID:19420309
Enterobacteria phage T4	Unconfirmed	*Klebsiella pneumoniae*	PMID:16936293
*Streptococcus pyogenes*	PMID:12019138	Hepatitis B virus F	PMID:34593159
*Candida tropicalis*	PMID:20455400	Human herpesvirus 5	PMID:32021322
*Klebsiella variicola*	PMID:30060219	*Candida albicans*	PMID:28409362
*Actinobacillus actinomycetemcomitans*	PMID:26538521	*Listeria ivanovii*	PMID:36981047
*Actinomyces oris*	Unconfirmed	*Marinobacter hydrocarbonoclasticus*	Unconfirmed

The microorganism that we have selected is *E. coli*, a conditionally pathogenic bacterium that under certain conditions can cause gastrointestinal infections or a variety of localised tissue and organ infections such as urogenital infections in humans and a wide range of animals [36]. Pathogenic *E. coli* can cause more than 16.01 billion cases of dysentery [37] and 1 million deaths annually, whereas non-pathogenic *E. coli* are part of the normal gut flora of healthy mammals and birds. For example, it is anticipated that the *E. coli* strain nissle will be utilized to cure human illnesses in addition to being utilized as a probiotic and therapeutic agent [38]. As shown in Table 5, 15 out of the top 20 predicted drugs have been confirmed by published journals to be associated with the *E. coli*.

**Table 5:** The top 20 forecasted drugs linked to *E. coli*. In this table, the first column lists the top 10 predicted drugs, while the third column lists the top 11 to 20 predicted drugs

Drug	Evidence	Drug	Evidence
(10R,11R)-Hydnocarpin	PMID:26273725	14-alpha-lipoyl andrographolide	PMID:19,652,378
3,5-Diiodotyrosine	PMID:36323433	2-(4,5-dibromo-1-methyl-1H-pyrrol-2-yl)-5-(2,4-dichlorophenyl)-1,3,4-oxadiazole	Unconfirmed
Cefditoren	PMID:17651945	3-[(prop-2-ene-1-sulfinyl)sulfanyl]prop-1-ene	Unconfirmed
Cefalonium	PMID:36065056	3,5-Dimethyl benzyl dodecyl beta-maltoside	Unconfirmed
para-Benzoquinone	PMID:27134027	Magainin-I	PMID:30,277,857
Hinokitiol	PMID:17927050	(1E)-1-{[(1E)-prop-1-ene-1-sulfinyl]sulfanyl}prop-1-ene	Unconfirmed
Hexameric peptide	PMID:20097816	Dicyclohexylamine	PMID:6,508,744
para-ethylaniline	PMID:28383815	(10R,11R)-Hydnocarpin D	PMID:26,273,725
3-{2-[(1S,2R,4aR,8aR)-1,2,4a,5-tetramethyl-1,2,3,4,4a,7,8,8a-octahydronaphthalen-1-yl]ethyl}-5-methylidene-N-phenyl-2,5-dihydrofuran-2-amine	Unconfirmed	3,4-Dichloro-cinnamaldehyde	PMID:27,939,874
hLF1-11	PMID:24631659	Paromomycin	PMID:60,235

## Conclusion and discussion

In this paper, we developed a new prediction model called GARFMDA by combining a two-layer GAT with a two-layer random forest to detect possible drug-microbe correlations. Results of both comparison experiments and case studies showed that GARFMDA exceeded these state-of-the-art competitive prediction models. Naturally, GARDFMDA can also be adopted to solve other problems involving the association prediction of biological entities, such as the prediction of associations between diseases and circRNA and microbes. Of course, GARFMDA can yet be improved. For instance, we can add more biological data, like microbial sequencing information, to the feature selection section [9]. Additionally, because the dataset is sparse, the model frequently results in the overfitting phenomena. To address this issue, we can also think about data augmentation. Moreover, the public database is not updated in real time, which may affect the way that the model is used in practice, therefore, we might consider to reconstruct an extensive database in the future.

### Supplementary Information

**Additional file 1**. The ID Numbers and Names of Newly-Downloaded Diseases.

**Additional file 2**. The ID Numbers and Names of Newly-Downloaded Drugs.

**Additional file 3**. The ID Numbers and Names of Newly-Downloaded Diseases.

**Additional file 4**. Newly-Downloaded Known Associations between Drugs and Diseases.

**Additional file 5**. Newly-Downloaded Known Associations between Drugs.

**Additional file 6**. Newly-Downloaded Known Associations between Drugs and mcrobes.

**Additional file 7**. Newly-Downloaded Known Associations between Microbes and Diseases.

**Additional file 8**. Newly-Downloaded Known Associations between Microbes.

## Data Availability

The original contributions presented in the study are included in the article, further inquiries can be directed to the corresponding authors.
